# Privacy Leaks Protection in Music Streaming Services Using an Intelligent Permissions Management System

**DOI:** 10.1155/2022/5027256

**Published:** 2022-06-08

**Authors:** Qian Wang

**Affiliations:** Zhengzhou Preschool Education College, Zhengzhou, Henan 450000, China

## Abstract

A security violation is referred to as a personal data breach when it leads to unintentional and unlawful destruction, loss, alteration, unauthorized disclosure, or access to personal data that has been communicated, stored, or otherwise processed in some other manner. Based on the principles of information security, we can define a breach of confidentiality as the unauthorized or accidental disclosure or access to personal data, a breach of integrity as the unauthorized or accidental alteration of personal data, and a breach of availability as the unauthorized or accidental loss of access to or destruction of personal data. This paper suggests designing an intelligent consensus policy management system based on the Markov chain approach. It is a novel system that would analyze the present status of the consensus elements for future development and anticipates the possibility of possible breaches of sensitive personal data. The evaluation of the proposed strategy is based on a policy scenario that involves a hypothetical consensus and a data breach of sensitive information to music streaming services.

## 1. Introduction

Online streaming replaces the purchase of Compact Disc-CDs, and podcasts come and take the place of radio. In recent years, more and more people are changing their habits in the music industry because we are in the middle of rapid development [1]. The ability to listen to music directly on the mobile via the Internet, wherever we are, surpasses in ease any other method. However, the sound and listening quality are not the same as traditional. For this very reason, streaming services were created from which we can legally listen to music [2]. Most of them are designed to help the user discover new music, with the software itself suggesting what new we will listen to based on our musical preferences. Of course, a prerequisite for these services is payment. After a short trial period that requires a credit card, a small monthly fee gives access to many songs.

However, the streaming era brings new issues, the most basic of which is the acquisition of sensitive personal data and its “processing” via automated means. Personal data are information about a living person who can be identified (e.g., name, home address, e-mail address, location data, and usage data) [3]. If put together, different information can identify a particular person as personal data. The term “processing,” by automated means as well as nonautomated processing includes the following activities: data collection and registration; organization and structure; storage; adaptation and modification; retrieval; retrieval of information; use; disclosure by transmission, dissemination, or any other form of disposal; correlation or combination; and restriction; deletion or destruction of personal data [4].

To be valid, personal data processing consent must be provided clearly and concisely, in language that is easy to understand and different from other information, such as terms and conditions. In addition, consent must be freely given, specific, aware, and unequivocal to be valid [5]. The request must state the purpose for which personal data will be used [6]. In certain circumstances, data subjects have the right to refuse to be subjected to a decision exclusively based on automated processing. Although this norm is generally followed, there are a few exceptions, such as when the data subject has explicitly consented to an automated decision-making mechanism. A data breach occurs when personal data are disclosed, accidently or illegally, to unauthorized recipients, made temporarily unavailable, or altered [7, 8].

So, it is essential to have an intelligent mechanism that can manage the consent granted to have a case for creating a profile, which can regulate the use, and therefore, there is a case of data leakage. Based on the adoption of the Markov chain methodology [9], it is proposed to implement an intelligent consent management system in Music Streaming Services, which considers the current state of consent for the future development and forecast possible leaks of sensitive personal data [3, 10].

## 2. Related Literature

This section explores relevant work on Markov chains implementation, privacy concerns, risk perception, and user behavior in response to security recommendations [11, 12]. Wiering et al. [13] investigated multiobjective Markov decision systems in 2007 by substituting a cross benefit vector for the conventional linear reward signal. This multiobjective Markov procedure may be transferred when the weighting factor for the various reward elements is known in advance. They anticipated that the weighting function might be arbitrarily chosen and given by the actor or user after the algorithm addressed the issue. They maintained track of Pareto's optimum stationary policies to cope with it.

Feinberg and Rothblum [14] investigated a Markov selection procedure with a different reward system and a specified beginning state dispersion. Suppose the habitation measure of a stationary policy can be described as a convex sum of the habitation evaluations of other stationary policies. In that case, the policy may be divided into states. There are requisite circumstances for dividing a stationary policy in a single state and adequate criteria for splitting it throughout the entire state area. The findings were used to limited issues to compute an optimum policy by calculating and dividing an ideal stationary policy.

O'Connor et al. [15] investigated the optimum transport issue for couples of stationary constraint Markov chains, focusing on calculating ideal transitional connections, a limited family of transfer plans that encapsulate the characteristics of Markov chains. The optimum changeover connectivity issue is solved by aligning the two chains to minimize the overall long-term price. They produced a stable conclusion for both normalized and nonregularized methods and, consequently, a probabilistic coherence result. They tested their theoretical predictions through a mock trial, indicating that the approximation technique has a shorter total runtime and a low error rate. Finally, they expanded their approaches to hidden Markov structures and demonstrated the suggested algorithms' practical use via the implementation of computer-generated music.

Concerning privacy leaks, Yixin et al. [7] conducted semistructured surveys with customers to ascertain their perceptions of the dangers associated with data breaches, their willingness to take preventive actions, and their motivations for inactivity. They discovered that users' mind maps of credit agencies were inadequate and erroneous to a certain extent. They discovered that this conduct is motivated by the expenditures of preventive methods, a positivity bias in evaluating one's chance of persecution, sources of guidance, and a general inclination to wait for response until damage occurs. They reviewed the legal, technological, and pedagogical ramifications and possible approaches for improving consumer protection in the credit reporting system. Finally, they suggest future research options.

Gwebu et al. [8] investigated the relative usefulness of corporate credibility and post-breach reaction techniques in light of the considerable monetary losses related to data intrusions. The findings suggested that a firm's brand is a critical asset for preserving the firm's worth. Nevertheless, only particular reaction tactics are shown to lessen the fiscal effect on low-reputation organizations. At the same time, reply techniques are less critical for high firms. These results provide operators with proof counsel for preserving business assets after privacy violations and emphasize the demand for establishing more sophisticated breach management techniques. The generated theoretical justifications provided a cognitive foundation for evaluating the effectiveness of different data breach response tactics.

## 3. Methodology

**Figure 1:**
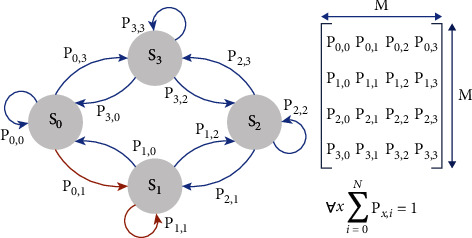
Markov chain.

The proposed methodology concerns implementing a policy consensus rights management system executed for each policy with a stationary Markov policy [9]. The Markov policy is considered to be a Markov chain {*Xn*(*R*)}*n* ∈ *ℕ*0 which is nondegradable or, more generally, has a unique closed communication class (meaning that transient states outside it are allowed), which is (genuinely) finite repetitive [16]. The idea of modeling is that Markov processes are appropriate stochastic models for describing and studying stochastic systems; the future evolution of which depends solely on their present state each time and not on their specific history. An example of a Markov chain and its mathematical modeling in predicting future situations is shown in Figure 1 [17–19].

Based on the adoption of the methodology of the Markov chains, it is proposed to implement an intelligent consensus policy management system, which considers the current state of the consensus data for the future development and forecasting of possible leaks of sensitive personal data [10]. A stochastic or Markov process [20–22] (or evolution) from *T* to *S* is a collection of random variables {*X*(*t*, *ω*)}_*t*∈*T*,*ω*∈Ω_ defined in a probability space (Ω, *F*, *P*), where *T*=*ℕ*_0_={0,1,2,…} the time horizon (usually *T* is the set of times). *S* is the state space of the process, i.e., the value field of (*t*, *ω*) for each *t* ∈ *T* and *ω* ∈ Ω. Because *S* is countable or finite, we are talking about a stochastic chain [23]. Ω is the sample space, i.e., the set of all possible results of the luck experiment under study. *F*: *σ*-algebra of contingencies is the field of definition of the contingencies of Ω (there are cases of sample space of Ω where we cannot consider each of its subsets as a possibility) and *P* is the probability measure, i.e., a function *P*: *F*⟶*ℝ* such that [21, 22]

1. *P*(Ω)=1
2. *P*(*A*) ≥ 0, ∀*A* ∈ ℱ
3. *P*(*U*_*V*=1_^*∞*^*A*_*v*_)=∑_*v*=1_^*∞*^*P*(*A*_*v*_), ∀{*A*_*v*_}_*v*∈*ℕ*_ which (*A*_*i*_∩*A*_*j*_=∅∀*i* ≠ *j*)

The proposed process focuses on a system of Markov chains, i.e., processes from Τ =  *ℕ* 0 in a countable (or simply finite) space *S*, which have the Markov property [24–26]

(1) $$\begin{array}{c} P\left(X_{n+1}=j|X_{0},X_{1},\ldots ,X_{n}\right)=P\left(X_{n+1}=j|X_{n}\right),\;n\in {\mathbb{N}}_{0},j\in S. \end{array}$$

The above relation states that given the value of the random variable *X*_*n*_ (present), the random variable *X*_*n* + 1_ (future) is stochastically independent of the variables *X*_0_, *X*_1_,…, *X*_*n* − 1_ (past state of the process) [27]. The explanation of the Markov property of the proposed system is that the future of the Markov chain depends on the past only through the present [28]. So, the probability [22, 24]

(2) $$\begin{array}{c} p_{ij}\left(n,n+1\right):=P\left(X_{n+1}=j|X_{n}=i\right), \end{array}$$

is the probability of passing 1st order from state *i* to state *j* by the (*n* + 1)-th step. These probabilities do not depend on the time step *n* (stationary), i.e., [29, 30]

(3) $$\begin{array}{c} p_{ij}=P\left(X_{n+1}=j|X_{n}=i\right)=P\left(X_{1}=j|X_{0}=i\right), \end{array}$$

so we have homogeneous chains {*Xn*}.

Respectively, considering the 1st order transition probability table of the chain [31–33],

(4) $$\begin{array}{c} \mathbb{P}={\left\{p_{ij}\right\}}_{i,j\in S}, \end{array}$$

we have a stochastic table, so it is easy to find the chain in the states *i*_0_, *i*_1_,…, *i*_*n*−1_, *i*_*n*_ ∈ *S* in succession; the probability is as follows:

(5) $$\begin{array}{c} P\left(X_{0}=i_{0},X_{1}=i_{1},\ldots ,X_{n-1}=i_{n-1},X_{n}=i_{n}\right)=\pi_{0}\left(j\right)\cdot p_{i_{0}i_{1}}\cdots p_{i_{n-1}i_{n}}. \end{array}$$

Thus, for the distribution *πn* of the state of the chain, it is valid that [28, 32]

(6) $$\begin{array}{c} \pi_{n}\left(j\right)=P\left(X_{n}=j\right)\forall j\in S. \end{array}$$

So,

(7) $$\begin{array}{c} \pi_{n+1}\left(j\right)=\sum_{i\in S}P\left(X_{n}=i\right)P\left(X_{n+1}=j|X_{n}=i\right)=\sum_{i\in S}\pi_{n}\left(i\right)\cdot p_{ij}, \end{array}$$

or equivalents

(8) $$\begin{array}{c} \pi_{n+1}=\pi_{n}\mathbb{P},\;\forall n\in {\mathbb{N}}_{0}, \end{array}$$

and so

(9) $$\begin{array}{c} \pi_{n}=\pi_{0}{\mathbb{P}}^{n}, \end{array}$$

where

(10) $$\begin{array}{c} {\mathbb{P}}^{n}={\left\{p_{ij}^{\left(n\right)}\right\}}_{i,j\in S}, \end{array}$$

the *n*-th order transition table (or *n* steps), i.e.,

(11) $$\begin{array}{c} p_{ij}^{\left(n\right)}=P\left(X_{n}=j|X_{0}=i\right). \end{array}$$

If we choose *π*0 = *π* such that *π* = *π*ℙ, we obtain *πn* = *π*∀*n*∈*ℕ*, i.e., the state distribution of the chain is stationary (independent of *n*). Then, the chain has an unchanged or stationary distribution, and it is in statistical equilibrium. In queuing theory, systems are said to be in “statistical equilibrium” when the number of customers or objects waiting in the line oscillates so that the mean and distribution remain the same over a prolonged period.

Therefore, to find the stationary distribution *π*, it is enough to solve the system [21, 28, 34, 35]:

(12) $$\begin{array}{c} \left\{\begin{array}{c} \sum_{i\in S}^{\pi =\pi \cdot P}\pi \left(i\right)=1,\\ \pi \left(i\right)\geq 0,\forall i\in S. \end{array}\right. \end{array}$$

A chain will be nondegradable if the state space *S* is an entire closed class, i.e.,

(13) $$\begin{array}{c} C\left(X_{n}=i,i\in C\right). \end{array}$$

The basic premise of the methodology is that the Markov chain

(14) $$\begin{array}{c} {\left\{X_{n}\right\}}_{n\in {\mathbb{N}}_{0}}, \end{array}$$

is nondegradable, genuinely repetitive, and aperiodic. Then, regardless of its initial distribution is valid,

(15) $$\begin{array}{c} \underset{n⟶\infty }{\lim}\pi_{n}=\pi , \end{array}$$

and

(16) $$\begin{array}{c} \underset{n⟶\infty }{\lim}p_{ij}^{\left(n\right)}=\pi \left(j\right),\;\forall i\in S, \end{array}$$

the percentage of time spent by the chain in each *i*∈*S* situation is *π*(*i*), so according to the employer theorem, we have

(17) $$\begin{array}{c} P\left[\frac{V_{n}\left(i\right)}{n}⟶\pi \left(i\right),\;\forall i\in S\right]=1. \end{array}$$

So, the transition from one state to another implies a fee or some cost (negative fee) of the form as follows:

(18) $$\begin{array}{c} R_{n}=R_{n}\left(X_{n-1},X_{n}\right),\;n\in \mathbb{N}, \end{array}$$

for the *n*-th step, so the total reward in the first *n* steps is

(19) $$\begin{array}{c} C\left(n\right)=\sum_{s=1}^{n}R_{s}\left(X_{s-1},X_{s}\right),\;n\in \mathbb{N}. \end{array}$$

The average fee for going *i* ⟶ *j* is

(20) $$\begin{array}{c} r_{ij}=E\left[R_{n}\left(i,j\right)\right]<\infty ,\;i,j\in S. \end{array}$$

Ultimately, it applies to the pay rate (average pay per step) as follows:

(21) $$\begin{array}{c} \underset{n⟶\infty }{\lim}\frac{C\left(n\right)}{n}=\sum_{j}r_{j}\cdot \pi_{j}. \end{array}$$

A fascinating question in the modeling of the proposed methodology concerns how we will choose the stationary policy or equivalent by what criteria this choice will be made. Since *C*(*i*, *α*) expresses sensitive data, we will deal with the standard of minimizing their propagation rate, which proves to be the most suitable in many applications.

Of course, if *C*(*i*, *α*) represents nonconsent of rights, the criterion will be maximizing the consent rate, which is equivalent to the previous one [36]. A stationary policy is *X*_0_ = *i*, *i*∈ S . Also *p*_*ij*_^(*k*)^(*R*) are the probabilities of passing *k*-th class [37]. Then, the average (or expected) total amount of consonants in the first *n* time points (or steps) when *X*_0_ = *i* and *R* is applied [18, 32, 38]:

(22) $$\begin{array}{c} V_{n}\left(i,R\right)=E_{R}\left[\sum_{k=0}^{n-1}C\left(X_{k},a_{k}\right)|X_{0}=i\right], \end{array}$$

or by the definition and linearity of the (bound) average value:

(23) $$\begin{array}{c} V_{n}\left(i,R\right)=\sum_{k=0}^{n-1}\sum_{j\in S}p_{ij}^{\left(k\right)}\left(R\right)\cdot C\left(j,R_{j}\right), \end{array}$$

or else

(24) $$\begin{array}{c} V_{n}\left(i,R\right)=E_{R}\left[\sum_{k=0}^{n-1}\lambda^{k}C\left(X_{k},a_{k}\right)|X_{0}=i\right]. \end{array}$$

Our objective is to study the behavior of the rate of the average rate of consensus in the long run, i.e., the limit as follows:

(25) $$\begin{array}{c} \underset{n⟶\infty }{\lim}\frac{V_{n}\left(i,R\right)}{n}, \end{array}$$

for each *i*∈ S.

However, because the Markov chain has a unique closed communication class (positively repetitive), the limit is unique and independent of the initial state *i* [39]:

(26) $$\begin{array}{c} g\left(R\right)=\underset{n⟶\infty }{\lim}\frac{v_{n}\left(i,R\right)}{n}\\ =\sum_{j\in S}\pi_{j}\left(R\right)\cdot C\left(j,R_{j}\right), \end{array}$$

with *π*_*j*_(*R*), *j*∈*S* is the stationary distribution of the chain below the policy *R* and thus the rate of change of average rate of consent or, in the long run, average rate per unit time. Therefore, a stationary policy *R*∗ will be optimal for the average consent rate if each stationary policy *R* applies [40]

(27) $$\begin{array}{c} g\left(R^{*}\right)\leq g\left(R\right). \end{array}$$

So, since the time horizon is unlimited and the space of situations is finite, it turns out that there will always be a stationary policy that will be optimal in terms of the above criterion. Considering this and using the proposed methodology for each possible stationary policy, we can calculate the stationary distribution and the corresponding rate in each case. The above result is significant because it secures the right to seek an optimal approach for the stationary.

## 4. Privacy Leaks Protection Scenario

To protect individuals' privacy and help restore trust and transparency in the activities between people and entities that process their data, streaming services provide consent. The data subject's permission is any freely given, precise, informed, and unequivocal expression of the data subject's desires by which he, by a statement or by an obvious action, accepts the processing of personal data about them. Consents are divided into “Free,” “Specific,” “Informed,” and “Indisputably indicated.” In the scenario tested by the proposed system, consents are implemented to process users' data. The policy concerns consent if at least all four of the following conditions apply [6, 41, 42]:

1. Free means that the data subjects are selected and controlled. It is invalid consent if the data subject does not have a natural option, is obliged to consent, or will suffer an undesirable consequence if they do not consent. Unless the consent is accompanied by a nonnegotiable provision of the terms and conditions, it has been begrudged.
2. Specifically, it aims to ensure user control and transparency for the data subject.
3. Informed aims to provide information to the persons to whom the data refer before their consent and is necessary to be able to make informed decisions, to understand what they agree on, and for example, to exercise their right to withdraw their consent.
4. Indisputably indicates that there should be no doubt that the data subject has agreed to the data processing [29, 43].

With the policy improvement method starting from any *R* policy, we check if it is optimal or not. If not, we find a policy *R*′ with *g*(*R*′) ≤ *g*(*R*) and check if it is optimal. Keeping in mind that the number of stationary policies is limited (on account of the fact that both the situation space and the decision space are limited), we can proceed with the procedure described above until we find the most effective policy.

The benefit of using this method is that rather than controlling all of the available stable policies, we only have to maintain a typically small fraction of them and progress from one strategy to another policy improvement.

The equation makes the beginning [44–46]:

(28) $$\begin{array}{c} g\left(R\right)=\underset{n⟶\infty }{\lim}\frac{V_{n}\left(i,R\right)}{n},\;\forall i\in S, \end{array}$$

which implies that asymptotically (i.e., for *n* ⟶ ∞: large *n*) holds

(29) $$\begin{array}{c} V_{n}\left(i,R\right)≅ng\left(R\right)+u_{i}\left(R\right),\;\forall i\in S. \end{array}$$

The quantities *u*_*i*_(*R*) are relative values of the states *i* when a stationary policy *R* is applied, and their difference is equal to

(30) $$\begin{array}{c} u_{i}\left(R\right)-u_{j}\left(R\right)≅V_{n}\left(i,R\right)-V_{n}\left(j,R\right),\;\forall i,j\in S. \end{array}$$

The relative values express the transient effect of the initial states on the expected total rate under the application of policy *R*.

Then, the quantity *hu*_*i*_(*R*) − *u*_*j*_(*R*) expresses the difference in the average total rate, if the process starts from the state *i* compared with whether it began to from state *j*, when the policy *R*.

Equivalently applied, this difference is essentially the maximum rate of consensus so that the system (the chain) starts from state *j* rather than *i* below state policy *R*.

If we assume an aperiodic chain *Xn*(*R*), then the limit exists [9, 14, 21]:

(31) $$\begin{array}{c} \underset{n⟶\infty }{\lim}p_{ij}^{\left(k\right)}\left(R\right)=\pi \left(j\right). \end{array}$$

So, there is also

(32) $$\begin{array}{c} \underset{n⟶\infty }{\lim}V_{n}\left(i,R\right)=\underset{n⟶\infty }{\lim}\sum_{k=0}^{n-1}\sum_{j\in S}p_{ij}^{\left(k\right)}\left(R\right)\cdot C\left(j,R_{j}\right). \end{array}$$

So,

(33) $$\begin{array}{c} u_{i}\left(R\right)-u_{j}\left(R\right)=\underset{n⟶\infty }{\lim}\left[V_{n}\left(i,R\right)-V_{n}\left(j,R\right)\right], \end{array}$$

which expresses the long-term difference in the mean total rate if the process starts from state *i* rather than state *j*, under policy *R*.

Therefore [29],

(34) $$\begin{array}{c} V_{n}\left(i,R\right)=C\left(i,R_{i}\right)+\sum_{j\in S}p_{ij}\left(R_{i}\right)\cdot V_{n-1}\left(j,R\right), \end{array}$$

and so

(35) $$\begin{array}{c} u_{i}\left(R\right)+g\left(R\right)≅C\left(i,R_{i}\right)+\sum_{j\in S}p_{ij}\left(R_{i}\right)\cdot u_{j}\left(R\right), \end{array}$$

or, respectively,

(36) $$\begin{array}{c} u_{i}\left(R\right)≅C\left(i,R_{i}\right)-g\left(R\right)+\sum_{j\in S}p_{ij}\left(R_{i}\right)\cdot u_{j}\left(R\right). \end{array}$$

So, for finding a rhythm and relative values based on the genuine iterative state *r*∈*S*, it holds [47]:

(37) $$\begin{array}{c} T_{i}\left(R\right)=1+\sum_{j\neq r}p_{ij}\left(R_{i}\right)\cdot T_{j}\left(R\right), \end{array}$$

where *T*_*i*_(*R*) is the expected time of the first visit to *r* since the chain started from state *i* under policy *R*.

For the average rate *K*_*i*_(*R*), the following applies:

(38) $$\begin{array}{c} K_{i}\left(R\right)=C\left(i,R_{i}\right)+\sum_{j\neq r}p_{ij}\left(R_{i}\right)\cdot K_{j}\left(R\right). \end{array}$$

Combining the above two equations, we take

(39) $$\begin{array}{c} K_{i}\left(R\right)-g\left(R\right)\cdot T_{i}\left(R\right)=C\left(i,R_{i}\right)-g\left(R\right)+\sum_{j\neq r}p_{ij}\left(R_{i}\right)\left[K_{j}\left(R\right)-g\left(R\right)\cdot T_{j}\left(R\right)\right],\\ u_{i}\left(R\right)=C\left(i,R_{i}\right)-g\left(R\right)+\sum_{j\neq r}p_{ij}\left(R_{i}\right)\left[K_{j}\left(R\right)-g\left(R\right)\cdot T_{j}\left(R\right)\right]+p_{ir}\left(R_{i}\right)\cdot u_{r},\\ u_{i}\left(R\right)=C\left(i,R_{i}\right)-g\left(R\right)+\sum_{j\neq r}p_{ij}\left(R_{i}\right)\cdot \left[K_{j}\left(R\right)-g\left(R\right)\cdot T_{j}\left(R\right)\right]+\\ \;+p_{ir}\left(R_{i}\right)\cdot \left[K_{r}\left(R\right)-g\left(R\right)\cdot T_{r}\left(R\right)\right],u_{r}=0. \end{array}$$

So, it finally applies that

(40) $$\begin{array}{c} u_{i}\left(R\right)=C\left(i,R_{i}\right)-g\left(R\right)+\sum_{j\in S}p_{ij}\left(R_{i}\right)\cdot \left[K_{j}\left(R\right)-g\left(R\right)\cdot T_{j}\left(R\right)\right], \end{array}$$

or else

(41) $$\begin{array}{c} u_{i}\left(R\right)=C\left(i,R_{i}\right)-g\left(R\right)+\sum_{j\in S}p_{ij}\left(R_{i}\right)\cdot u_{j},\;i\in S, \end{array}$$

namely,

(42) $$\begin{array}{c} \left(g,\underline{u_{i}}\right)=\left(g\left(R\right),\underline{u_{i}\left(R\right)}\right), \end{array}$$

are a solution of the original system, and therefore the request was proved.

## 5. Conclusions

Inside the scope of this study, we suggested a novel policy-based consensus management system intending to prevent data breaches within music streaming services. The methodology that has been proposed is solely founded on an advanced Markov chain system. This creates an intelligent consensus policy management framework that considers the current state of the consensus data to predict potential leaks of sensitive personal data and prepare for their development in the future. To be more specific, we employ Markov processes with a discrete (limited or countable) state space and a distinct parametric space. We examine this way of improvement to see whether or not a policy is optimum. It is a forward-thinking and clever system that can model challenging scenarios, making it virtually more straightforward to discover answers to questions regarding dynamic circumstances of ambiguity.

The provision of consent by streaming services helps to reestablish confidence and transparency in the interactions between individuals and the organizations responsible for processing their data. This safeguards the privacy of individuals. The data subject's permission is any freely given, precise, informed, and unambiguous expression of the data subject's desires by which he, by a statement or by an obvious action, accepts the processing of personal data about them. This expression of the data subject's desires can take the form of a statement or an apparent effort. The terms “Free,” “Specific,” “Informed,” and “Indisputably indicated” are used to classify different types of consent. In the hypothetical situation examined by the suggested system, permissions are successfully applied to handle users' data successfully.

The proposed tactic, in addition to the apparent advantages, lags because of the increased complexity even for a small space of solutions S. For example, for a set of *N* with two possible decisions (the same for each situation), from the simple multiplication principle, we have 2*N* different stationary policies. For *N* = 10, we have a total of 1024 stationary policies. We can overcome this obstacle with optimization methods and selecting a predefined solution space. Therefore, significant future development of the proposed system is the investigation of optimization methods using biologically inspired methods to find the optimal solution spaces that could significantly simplify the proposed methodology.

## Data Availability

The data used in this study are available from the author upon request.
